# Explanation of a Statement Contained in Mr. Atkinson's Paper*

**Published:** 1817-02

**Authors:** William Hey


*For the London Medical and Physical Journal.*

### *Explanation of a Statement contained in Mr. Atkinson's* Paper;* by WILLIAM HEY, Junior.

AFTER the intimations of your wish, contained in your two last Numbers, that the controversy on obstetric practice might cease, I would not have troubled you with any remarks on that subject, had I not feared that a statement of cases in midwifery, with which I furnished Mr. Atkinson, might make an erroneous impression on the minds of your readers. I beg your permission, therefore, so far to explain that statement, as to prevent or counteract the misconstruction to which it is liable.

The misconstruction to which I refer might, perhaps, partly arise from a want of precision in the statement itself, and partly from the manner in which Dr. Kinglake has noticed it. From various passages in his last reply to Mr. Atkinson, he appears to consider it as a fair inference from the statement, and as one intended to be drawn from it, "that about *one* in *five* cases of human parturition would be *unnatural.*" I am far from entertaining such an opinion; and, I doubt not, Mr. Atkinson is equally so. If the statement of my cases be duly attended to, with such additional explanation as I am now desirous to give, it will not be found to warrant a conclusion so alarming and unfounded.

Mr. Atkinson expressly mentions, that a number of the bad cases in question "(probably not less than thirty)" were attended in consultation with other practitioners. To this circumstance no allusion has been made, though it is obvious that all such cases must be excluded from any calculation respecting the proportion of *unnatural* cases in my practice. I stated this number according to the best of my recollection; but it was given partly from memory, as a distinction is not always made, in my minutes, between the cases of my own patients and those of others. I am pretty confident it was not less; it might be more.

Again, Dr. Kinglake says, "Mr. Atkinson may call on the younger Mr. Hey to report 150 unnatural cases out of 827 labours, &c." I must here observe, that Mr. A. does not call them "*unnatural cases:*" his expression is, that "150 were such as to require manual aid, either with regard to the expulsion of the placenta or child." Now, (besides the deduction to be made of the cases attended in consultation,) in fifty-two of this number, either it was necessary to

* See London Med. and Phys. Journal for August.

separate separate the placenta with the hand *in utero*, or it was thought adviseable, on account of too copious a discharge, occasioned by a partial separation of the placenta, to extract it quickly by pulling at the *funis*. If either of these circumstances happened after labours otherwise natural, I should think it improper to denominate the case *unnatural*, especially with regard to the latter kind of assistance (which was far the more frequent of the two), though it might have been greatly conducive to the welfare of the patient.

The term—unnatural case, ought to have been defined before I am charged with reporting "150 unnatural cases out of 827 labours." It would be easy to show, that cases of unnatural presentation are confounded with those requiring the aid of instruments; but I wish to avoid every thing that may not be strictly called explanation. I allude to this circumstance, in order to introduce the remark, that a small proportion of my 150 cases were of a kind to require the use of instruments. As Mr. Atkinson has not mentioned the number, it may be proper for me to state that they were thirty-three (in three of which, only one blade of the forceps was used); and I suppose that at least half of them were the patients of other practitioners with whom I attended in consultation.

It may not, perhaps, be thought too trivial to mention, that ten of the 150 were cases of twins; and, as one of the children might be born in the natural way, while the other might require manual assistance or instrumental delivery, it would seem proper to reckon each case of twins as two cases. If this were done in respect to all the twin cases of the 827 labours, it would still further diminish the proportion of bad cases.

After all, it may probably be thought, that (independently of consultations) I have met with an unusual proportion of difficult labours; and I am disposed to think that this has really been the case. So far as this is the fact, my practice will be an unfair criterion of the relative proportion of difficult and natural labours. Mr. Atkinson, indeed, has not drawn any inference from my statement respecting such proportion in general practice; and I have his authority for stating, that he intended no more than to show (what, after all reasonable allowances for peculiar circumstances) the statement does show) that cases requiring manual or instrumental aid, far exceed Dr. Kinglake's estimate.

From this brief explanation of the statement of my cases, I trust it will appear, that the conclusion drawn by Dr. Kinglake ("that about *one* in *five* cases of human parturition would be *unnatural*") is not fairly deducible from that statement.

ment. Certainly no such conclusion was intended by me. What Dr. Kinglake means by "suspecting the accuracy of my observations," I cannot tell. The minutes of my cases were made at the time they happened, and it is almost impossible there should be any inaccuracy as to the cases of unnatural presentations,* of presentations of the placenta, of puerperal convulsions, or of hydrocephalus. If he suspects (after the explanation now given) that I have had recourse to the use of instruments before the natural powers had had a sufficient trial,—to such a suspicion I can only oppose my own declaration, that I have so great an aversion to the use of instruments, that I believe myself to be in more danger of deferring their use too long than of employing them prematurely.

*Dec.* 1816.

* One exception may be made to this assertion, viz. that the distinction is not always clearly made, in my minutes, between cases of foot presentation and those in which it was judged right to deliver by the feet, before the presenting part was manifest, as might appear in some of the twin cases.

